# Progress and Challenges in the Design and Clinical Development of Antibodies for Cancer Therapy

**DOI:** 10.3389/fimmu.2017.01751

**Published:** 2018-01-04

**Authors:** Juan C. Almagro, Tracy R. Daniels-Wells, Sonia Mayra Perez-Tapia, Manuel L. Penichet

**Affiliations:** ^1^GlobalBio, Inc., Cambridge, MA, United States; ^2^Division of Surgical Oncology, Department of Surgery, David Geffen School of Medicine, University of California, Los Angeles, Los Angeles, CA, United States; ^3^UDIBI, Instituto Politécnico Nacional, México DF, México; ^4^Department of Microbiology, Immunology, and Molecular Genetics, David Geffen School of Medicine, University of California, Los Angeles, Los Angeles, CA, United States; ^5^Jonsson Comprehensive Cancer Center, University of California, Los Angeles, CA, United States; ^6^The Molecular Biology Institute, University of California, Los Angeles, CA, United States; ^7^UCLA AIDS Institute, Los Angeles, CA, United States

**Keywords:** therapeutic antibodies, oncology, humanization, chimerization, phage display, Fc engineering, transgenic mice

## Abstract

The remarkable progress in engineering and clinical development of therapeutic antibodies in the last 40 years, after the seminal work by Köhler and Milstein, has led to the approval by the United States Food and Drug Administration (FDA) of 21 antibodies for cancer immunotherapy. We review here these approved antibodies, with emphasis on the methods used for their discovery, engineering, and optimization for therapeutic settings. These methods include antibody engineering *via* chimerization and humanization of non-human antibodies, as well as selection and further optimization of fully human antibodies isolated from human antibody phage-displayed libraries and immunization of transgenic mice capable of generating human antibodies. These technology platforms have progressively led to the development of therapeutic antibodies with higher human content and, thus, less immunogenicity. We also discuss the genetic engineering approaches that have allowed isotype switching and Fc modifications to modulate effector functions and bioavailability (half-life), which together with the technologies for engineering the Fv fragment, have been pivotal in generating more efficacious and better tolerated therapeutic antibodies to treat cancer.

## Introduction

The hybridoma technology developed in the mid-1970s by Köhler and Milstein (1) proved to be an efficient means to isolate single specificity antibodies and produce them in unlimited amounts. This seminal achievement paved the way to effectively generate antibodies for a diverse array of therapeutic applications (2). Due to their exquisite specificity and high affinity, monoclonal antibodies have been considered particularly attractive molecules for diagnosis and/or therapy of multiple diseases, and currently antibody-based drugs represent the fastest-growing segment of all the therapeutic proteins in the biotechnology industry (3).

The first approved monoclonal antibody by the United States Food and Drug Administration (FDA) in 1985 (4) for therapeutic settings was muromonab-cluster of differentiation 3 (CD3) (Orthoclone OKT3^®^). This mouse monoclonal IgG2a antibody developed using the hybridoma technology, blocks CD3-mediated activation of T cells and was instrumental in the prevention of organ rejection after transplantation (5). Nonetheless, patients who were given Orthoclone OKT3^®^ developed a significant percentage of anti-drug antibodies, also known as a “human anti-mouse antibody” (HAMA) response (6). The HAMA response leads to the inactivation and elimination of the murine antibody (7). It also prevents the use of multiple administrations of the antibody that is required for the therapy of cancer. These issues, along with the fact that murine monoclonal antibodies can be associated with the generation of severe allergic reactions further hampered the use of antibodies of murine origin in human therapy (7).

Additionally, murine antibodies poorly interact with the human immune effector system. Relevant antibody effector functions mediated by the mouse fragment cyrstallizable (Fc), such as antibody-dependent cell-mediated cytotoxicity (ADCC), are decreased or absent in humans (8). This also applies to the interaction with the neonatal receptor (FcRn), also known as the Brambell or “*salvage receptor*”, which could result in a very short half-life of murine antibodies when used for human therapy (9). Hence, the multiple drawbacks of murine monoclonal antibodies as biotherapeutics in humans motivated efforts to make them more human-like molecules.

To engineer more human-like antibodies and, thus, increase efficacy while decreasing immunogenicity, non-human variable (V) domains were combined with human constant (C) domains to generate molecules with 70% or more human content. This method called chimerization was developed at the beginning of the 1980s (10) and led to the approval in 1997 of first chimeric therapeutic antibody to treat cancer, **rituximab** (Rituxan^®^). **Rituximab** has been a tremendous medical and commercial success, currently being the fourth best-selling innovative drug of any kind (3).

In parallel to the clinical development and success of **rituximab**, other technology platforms emerged in the 1980s and 1990s aiming to generate more human-like V domains. These technology platforms have been perfected during the last three decades and include humanization (11), selection of fully human antibodies from Fv and Fab phage-displayed libraries (12), and the development of transgenic animals capable of generating fully human antibodies (13, 14). Moreover, the ground-breaking work on chimerization (10) also highlighted the possibility of linking any V fragment to diverse human Fc isotypes to increase or decrease cytotoxicity. Since antibody effector functions such as ADCC have been considered important mechanisms of action (MOA) for cancer immunotherapy, human IgG1 was the isotype of choice for therapeutic development of the first approved oncology therapeutic antibodies (15). More recently, other isotypes such as human IgG2 and IgG4 have increasingly been used for therapeutic development in oncology. The first human IgG2 approved in 2006 to treat cancer was **panitumumab** (Vectibix^®^) (16). The MOA of **panitumumab** mostly relies on the target blockade rather than engaging immune effector killing mechanisms such as ADCC.

Discovery and optimization platforms to generate highly specific V regions with a higher human content for therapeutic settings combined with Fc engineering have enabled the approval of 21 antibodies to treat cancer (Table 1). This review focuses on these antibodies, lessons learned from their engineering and clinical development, as well as challenges and prospects to generate more efficacious therapeutic antibodies. We first provide an overview of the IgG molecule, the therapeutic format of the currently approved naked antibodies. Second, we briefly review the oncology targets for which there is more than one approved therapeutic antibody. Third, we discuss the human content of the approved antibodies and the technology platforms used to engineer their V regions. Finally, we provide a summary of the variations of effector functions and bioavailability (half-life) of human IgG isotypes and the approaches used to modify them. Since the Fc engineering field has achieved significant progress in the last few years, beyond the development and approval of the currently marketed antibodies, we also expand on Fc variants in study and/or clinical development.

**Table 1:** United States FDA-approved therapeutic antibodies to treat cancer as of July 30, 2017.

International non-proprietary names (INN)	Commercial name	Company	Approval date	Type	Isotype	Target	Indication
**Rituximab**	Rituxan^®^	Genentech	11/26/1997	Chimeric	IgG1	CD20	B-cell non-Hodgkin lymphoma
**Trastuzumab**	Herceptin^®^	Genentech	9/25/1998	Humanized	IgG1	HER2	Metastatic breast cancer
**Alemtuzumab**	Campath^®^	Genzyme	5/7/2001	Humanized	IgG1	CD52	B-cell chronic lymphocytic leukemia
**Cetuximab**	Erbitux^®^	ImClone Systems	2/12/2004	Chimeric	IgG1	EGFR	Metastatic colorectal carcinoma
**Bevacizumab**	Avastin^®^	Genentech	2/26/2004	Humanized	IgG1	VEGF	Metastatic colorectal cancer
**Panitumumab**	Vectibix^®^	Amgen	9/27/2006	Fully human	IgG2	EGFR	Metastatic colorectal cancer
**Ofatumumab**	Arzerra^®^	Glaxo Grp	10/26/2009	Fully human	IgG1	CD20	Chronic lymphocytic leukemia
**Ipilimumab**	Yervoy^®^	Bristol-Myers Squibb	3/25/2011	Fully human	IgG1	CTLA-4	Metastatic melanoma
**Pertuzumab**	Perjeta^®^	Genentech	6/8/2012	Humanized	IgG1	HER2	Metastatic breast cancer
**Obinutuzumab**	Gazyva^®^	Genentech	11/1/2013	Humanized	IgG1	CD20	Chronic lymphocytic leukemia
**Ramucirumab**	Cyramza^®^	Eli Lilly	4/21/2014	Fully human	IgG1	VEGFR2	Gastric cancer
**Pembrolizumab**	Keytruda^®^	Merck	9/4/2014	Humanized	IgG4	PD-1	Metastatic melanoma
**Nivolumab**	Opdivo^®^	Bristol-Myers Squibb	12/22/2014	Fully human	IgG4	PD-1	Metastatic melanoma
**Dinutuximab**	Unituxin^®^	United Therapeutics	3/10/2015	Chimeric	IgG1	GD2	Pediatric high-risk neuroblastoma
**Daratumumab**	Darzalex^®^	Janssen Biotech	11/16/2015	Fully human	IgG1	CD38	Multiple myeloma
**Necitumumab**	Portrazza^®^	Eli Lilly	11/24/2015	Fully human	IgG1	EGFR	Metastatic squamous non-small cell lung carcinoma
**Elotuzumab**	Empliciti^®^	Bristol-Myers Squibb	11/30/2015	Humanized	IgG1	SLAMF7	Multiple myeloma
**Atezolizumab**	Tecentriq^®^	Genentech	5/18/2016	Humanized	IgG1	PD-L1	Bladder cancer
**Olaratumab**	Lartruvo^®^	Eli Lilly	10/19/2016	Fully human	IgG1	PDGFRA	Soft tissue sarcoma
**Avelumab**	Bavencio^®^	EMD Serono	3/23/2017	Fully human	IgG1	PD-L1	Metastatic Merkel cell carcinoma
**Durvalumab**	Imfinzi^®^	AstraZeneca	5/1/2017	Fully human	IgG1	PD-L1	Urothelial carcinoma

*The table was generated by parsing the information on approved antibodies compiled by The Antibody Society (http://www.antibodysociety.org/news/approved-antibodies/), CenterWash (http://www.centerwatch.com/drug-information/fda-approved-drugs/), and contrasted with recent reviews on the state of the art in therapeutic antibodies for treatment of cancer cited in this review. The antibodies are listed chronologically in the table in the order of approval date. The INN are highlighted in bold in the Table and text of the article*.

It should be noted that in addition to naked antibodies, other therapeutic modalities to treat cancer based on the antibody molecule have been gaining momentum in recent years. Such modalities include antibodies conjugated to cytotoxic organic compounds, also known as antibody–drug conjugates (ADCs) (17) as well as antibodies conjugated to radionuclides (18), protein toxins (19), and immunomodulators such as cytokines (20). Other modalities known as bispecific antibodies (21), combining two specificities in a single molecular entity, have also shown increased efficacy and/or a novel MOA when compared to the combination of the two naked antibodies binding individual targets used as the source to engineer the bispecific molecule.

In fact, the relatively recent FDA approval of three ADCs (17) and two bispecifics (21) has fueled the engineering and clinical development of these modalities. The first FDA-approved ADC was gemtuzumab ozogamicin (Mylotarg™) for the treatment of acute myeloid leukemia (AML). Mylotarg™ was voluntarily withdrawn in 2010 in the United States market but, due to the critical unmet need of treatment for patients with AML, it has recently been reintroduced in the United States with different dosing and administration schedules. The two other FDA-approved ADCs are brentuximab vedotin (Adcetris^®^) and trastuzumab emtansinem (Kadcyla^®^), which have proven to be highly efficacious with limited toxicity (17). The two approved bispecific antibodies are catumaxomab (Removab^®^) and blinatumomab (Blincyto^®^). These antibody-based drugs have shown to be a breakthrough in the field of cancer immunotherapy. Both bispecifics bind CD3 on T-cells with one arm of the molecule. With the other arm, catumaxomab and blinatumomab bind cancer cells expressing epithelial cell adhesion molecule (EpCAM) or cluster of differentiation 19 (CD19), respectively. Simultaneous binding of CD3 and EpCAM or CD19 bring in the close proximity cancer cells with T-cells leading to a specific and highly efficacious killing process of the cancer cells (21). Although ADC and bispecific modalities are not reviewed here due to the vast amount of information published in this field, compounded with space limitations, it should be highlighted that the methods for discovery and optimization of V regions and modifications of the Fc to tailor the effector functions to a given MOA, are common and can be applied to all antibody-based modalities.

## The IgG Molecule

**Figure 1:**
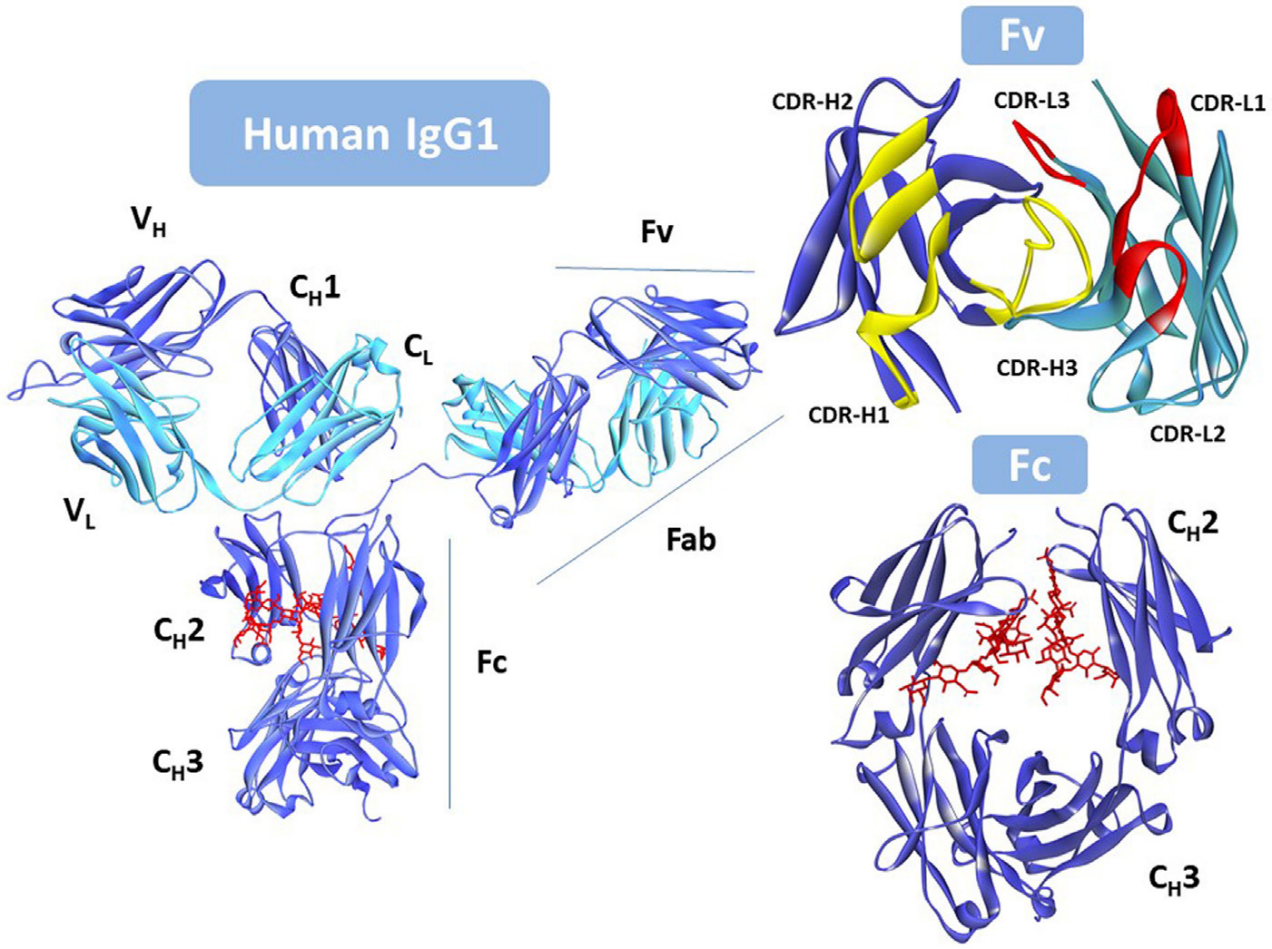
Intact human IgG1, Protein Data Bank (PDB) ID: 1HZH (23). Heavy chain is shown in blue. Light chain in cyan, and the *N*-glycan in red. Fv (top right) with the antigen-binding site seen from the antigen perspective. V_L_ complementarity-determining regions (CDRs) in yellow; V_H_ CDRs in red. Fc (bottom right) rotated with respect to the antibody to better show the location of the *N*-glycan (in red). Notice that one of the hinge peptides is missing in the figure. Due to its flexibility it was not solved since coordinates for this region are not available in the PDB file. This figure was generated using Discovery Studio.

Five different antibody classes exist in humans, determined by the nature of the C regions of the heavy chain (HC). These classes are designated by lower-case Greek letters: γ for IgG; δ for IgD; ε for IgE; α for IgA, and μ for IgM (22). The IgG is the most prevalent class of antibodies in blood and the most common molecular format used as therapeutic. Figure 1 shows that the IgG is assembled with two identical HCs and two identical light chains (LCs), classified in two types, κ and λ. The LC has a single variable (V_L_) domain and a single constant (C_L_) domain, whereas, the HC consists of a single variable (V_H_) domain, a hinge region, and three constant (C_H_1, C_H_2, and C_H_3) domains. The C_H_3 domain is located at the C-terminus of the IgG. In the N-terminus, the pairing of the LC and the Fd fragment (V_H_ and C_H_1) from the HC forms the fragment antigen binding (Fab), where the antigen-binding site is located. The heterotetrameric structure of IgG is held together covalently by disulfide bonds between the C_L_ and C_H_1 domains and between the hinge (interdomain) region of the two HCs.

The diversity of the antigen-binding site, and hence the capacity of antibodies to bind virtually any target, comes from diverse germline gene repertoires (24). The IGLV and IGLJ germline genes encode the V_L_ domain, whereas the V_H_ domain is encoded by the repertoires of IGHV, IGHD, and IGHJ germline genes (25). Additional amino acid variation in the antigen-binding site occurs through somatic mechanisms, such as somatic hypermutation in humans and mice, and gene conversion in other species such as chickens and rabbits (26, 27).

The germline and somatic amino acid variability is concentrated in the complementarity-determining regions (CDRs). Three CDRs in V_L_: CDR-L1, CDR-L2, and CDR-L3, and three in V_H_: CDR-H1, CDR-H2, and CDR-H3, alternate with conserved regions called framework regions (FRs), four in V_L_: FR-L1, FR-L2, FR-L3, and FR-L4, and four in V_H_: FR-H1, FR-H2, FR-H3, and FR-H4. The six CDRs are brought together by folding and non-covalent association of the V domains in the Fv (Figure 1) at the tip of the Fabs.

Two Fabs are linked to one Fc *via* the hinge region that provides flexibility to the antibody molecule to interact with diverse configurations of the targets. The Fc is formed by the non-covalent association of C_H_2 and C_H_3 domains, with critical residues in the hinge and C_H_2 determining the immune effector functions of the IgG antibody *via* interaction with the Fc gamma family of receptors (FcγRs) and the complement component C1q. Engagement of FcγRs on immune effector cells activates cellular responses such as ADCC and antibody-dependent cell-mediated phagocytosis (ADCP). Complement fixation, starting with the interaction of the antibody and the complement component C1q, induces activation and formation of the membrane attack complex (MAC), finally resulting in complement-mediated cytotoxicity (CDC).

The human IgG has four subclasses: IgG1, IgG2, IgG3, and IgG4, also known as isotypes (28). These isotypes have evolved different Fc sequences with differential capacity to elicit effector functions (Table 2). Isotype-specific engagement of such immune functions is based on selective Fc receptor interactions on distinct immune cell populations such as natural killer (NK) cells, neutrophils, and macrophages, as well as the ability to bind C1q, an initial protein in the complement pathway leading to a “cascade” of events that results in the formation of the MAC and the induction of tumor cell killing.

**Table 2:** Functional properties of the human IgG isotypes.

Properties	IgG1	IgG2	IgG3	IgG4
Approximate molecular weight (kDa)	146	146	165	146
Hinge length (number of amino acids)	15	12	62	12
Antibody-dependent cell-mediated cytotoxicity	+++	+/−−	++	+/−−
Antibody-dependent cell-mediated phagocytosis	+	+	+	+/−−
C1q binding	+	+/−	+++	–
Complement-mediated cytotoxicity	++	+/−	++	–
FcRn binding	+	+	+/−	+
Plasma half-life (days)	21	21	5–7.5	21
Approximate average plasma concentration (mg ml^−1^)	9	3	1	0.5

*Adapted from Strohl and Strohl (29) and Bruggemann et al. (30)*.

Additionally, IgG antibodies contain an *N*-glycosylation site at asparagine-297 (N297) in the C_H_2 domain. Modification of this *N*-linked glycan affects the Fc-mediated effector functions. Furthermore, specific residues located near the C_H_2–C_H_3 junction engage the major histocompatibility complex (MHC) class I related receptor, known as the FcRn that largely determines the blood half-life of antibodies.

## Therapeutic Antibodies Approved for the Treatment of Cancer

**Figure 2:**
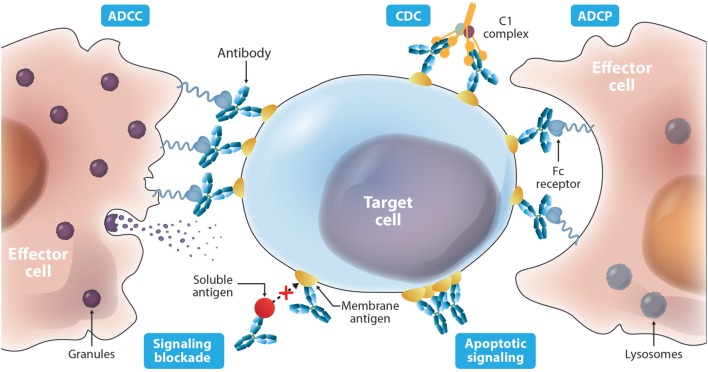
Diverse mechanisms of actions described for antibody-based drugs. Antibodies such as IgG1 can activate immune effector functions such as antibody-dependent cell-mediated cytotoxicity (ADCC), antibody-dependent cell-mediated phagocytosis (ADCP), and complement-mediated cytotoxicity (CDC) *via* specific binding to membrane targets on the cancer cell and binding to the Fc receptors on the surface of effector cells. Antibodies can also elicit protective activity by targeting a soluble ligand or their receptors on the surface of cancer cells blocking their interaction. In addition, targeting a cell surface receptor may trigger events that result in cytotoxic activity independent of blocking its ligand, such as receptor dysfunction due to cross-linking.

The current FDA-approved antibodies to treat cancer (Table 1) target 13 molecules including membrane proteins such as cluster differentiation 20 (CD20) and epidermal growth factor receptor (EGFR), soluble protein ligands for instance vascular endothelial growth factor (VEGF), and a disialoganglioside (GD2). These antibodies aim at different MOAs (Figure 2), which result from an interplay of the biology of the target, affinity of the antibody for the target, and/or effector functions such as ADCC, ADCP, and/or CDC that are elicited. The MOAs may also include blockade of oncogenic pathways with inhibition of malignant cell proliferation and/or induction of apoptosis, blockade of the formation of new blood vessels, and enhancement of the antitumor cytotoxic T cell (CTL) immune response to target tumor cells by inhibiting the immune cell checkpoint resulting in their activation.

The most relevant targets in terms of number of approved therapeutic antibodies are CD20, EGFR, its paralog human EGFR2 (HER2), and programmed cell death protein 1 (PD-1) and its ligand 1 (PD-L1). A brief description of these targets and the interaction with the therapeutic antibodies follows. A recent review on the above targets, other targets, and their interaction with therapeutic antibodies and MOAs has been published (31).

### Anti-CD20 Antibodies

Targeting CD20 with antibodies led to the approval of **rituximab** back in 1997, the first therapeutic antibody approved to treat cancer. CD20 is highly expressed on B cells throughout their development, but is absent on the hematopoietic stem cell (32). Although the physiological function of CD20 remains unclear, several lines of evidence suggest a role for CD20 in calcium signaling of B-cell antigen receptor activation. It has also been suggested that CD20 exists predominantly as a tetramer on the cell surface. CD20 is not shed or internalized upon antibody binding, which facilitate the recruitment of immune effector cells and mediate sustained immunologic activity as relevant MOA (33).

CD20 has four transmembrane domains with two extracellular loops, one large loop of 45 amino acids, and a short loop of nine residues. Anti-CD20 antibodies are classified as Type I or Type II according to their interaction with CD20 and primary MOA (32). **Rituximab** and **ofatumumab** are Type I antibodies, whereas **obinutuzumab** is a Type II. Among other characteristics, Type I antibodies have full binding capacity, high CDC, and moderate direct cell death induction. Type II antibodies have half binding capacity, low CDC, and stronger direct cell death induction.

Peptide scanning and mutagenesis studies have shown that **rituximab** binds the large CD20 loop (34). Although **obinutuzumab** is a Type II antibody, its epitope overlaps with that of **rituximab**, but is shifted toward the C-terminus of the large CD20 loop. X-ray crystallography (35) of the extracellular large loop in complex with **rituximab** or **obinutuzumab** Fabs have confirmed that while these antibodies bind partially overlapping epitopes, they differ in their interaction with the large CD20 loop.

**Ofatumumab** is a Type I antibody like **rituximab** but binds the small CD20 extracellular loop (32). Binding of **ofatumumab** seems to influence the large loop conformation but does not interact with the critical residues of CD20 determining the epitope of **obinutuzumab** and **rituximab**. The differences in primary MOA of **ofatumumab**, **obinutuzumab**, and **rituximab** suggest that in addition to the different epitopes they bind, other factors such as orientation of the antibodies when bound to CD20 are important in their therapeutic efficacy (32).

### Anti-EGFR Antibodies

EGFR and HER2 were among the first receptors to be identified and associated with human tumors (36, 37). Physiologically, EGFR (also known as HER1/ErbB1) and the EGFRs 2, 3, and 4 (HER2/ErbB2, also known as the *neu* oncogene, HER3/ErbB3, and HER4/ErbB3) are involved in cell growth control and differentiation. Several crystal structures of EGFR and HER2 and complexes with therapeutic antibodies are now available (38). The extracellular domain (ECD) of EGFR is composed of four domains I–IV, which are arranged in two conformations: an “extended” active form and the alternative inactive form, which is folded over or “tethered.” In the inactive form, domain II interacts with domain IV, while domains I and III are far apart. The active extended dimeric form is induced by the ligand, epidermal growth factor (EGF), in which domains I and III are closer together.

All three approved anti-EGFR therapeutic antibodies bind domain III and block the interaction with EGF. The X-ray crystal structures of **necitumumab** and **cetuximab** Fabs in complex with EGFR indicate that these antibodies bind a very similar surface on EGFR but, having different CDRs, do so through a set of different interactions (39). In fact, **necitumumab** was isolated from a human antibody phage-display library by competition with **cetixumab**. The similarity in the epitopes of **necitumumab** and **cetuximab** suggested that the former would have similar properties to the chimeric antibody **cetuximab**, but with the benefits of a fully human antibody (39).

The epitope recognized by **panitumumab** also overlaps with **cetuximab** (40). However, screening of peptide phage-display libraries and mutagenesis studies have shown that although these antibodies bind overlapping regions on EGFR, some amino acids in the epitope are critical for **cetuximab** binding, whereas others are specific for **panitumumab**. The relevance of these specific interactions in clinical settings emerged from studies in a patient with colorectal cancer who acquired a point mutation under treatment with **cetuximab** and developed resistance to treatment with this antibody, whereas treatment with **panitumumab** was still effective in this patient (41). This mutation seemed to abrogate **cetuximab** binding to the mutated EGFR, while **panitumumab** binding remained unaffected. Thus, differences in the functional epitopes of **panitumumab** and **cetuximab** could have clinical relevance as they may be instrumental in the selection of patients and decisions regarding their treatment.

### Anti-HER2 Antibodies

The structure of HER2 is similar to that of EGFR (42), but HER2 does not bind a ligand, functioning primarily *via* heterodimerization with ligand-bound partners of the EGFR family, mostly HER3. Comparison of several X-ray crystal structures (38) indicate that HER2 ECD adopts an extended conformation due to two non-conservative key mutations in the domain IV residues, which replace glycine 563 (G563) and histidine 565 (H565) in EGFR by proline (P) and phenylalanine (F) in HER2. These mutations prevent the contacts of domain II–IV, rendering in HER2 the extended active conformation seen in EGFR but without ligand binding in HER2. Moreover, HER2 ECD does not homodimerize in solution, perhaps due to conformational differences between the extended ECD module of HER2 and the EGFR dimeric conformation (38).

**Trastuzumab** binds domain IV, close to the membrane, and its MOA involves disruption of HER2 homodimerization and prevention of cleavage of the ECD, which leads to the active truncated receptor p95HER2 (43). This truncated form of HER2 maintains kinase activity and can migrate to the nucleus to act as oncogenic nuclear factor. **Pertuzumab** binds domain II and prevents heterodimerization of HER2 with HER3 and EGFR, blocking growth of HER2-amplified breast cancer (44). Of note, the combination of **pertuzumab** and **trastuzumab** in breast cancer therapy has been shown to be more efficacious than the treatment with the single therapeutics (45).

### Anti-PD-1/PD-L1 Antibodies

Programmed cell death protein 1 (PD-1) and its ligand (PD-L1) are immune checkpoints that inhibit CTL activity (46, 47). PD-L1 is constitutively expressed on a subset of macrophages, but may be rapidly upregulated in different tissue types and by tumors in response to interferon-gamma (IFN-γ) and other inflammatory mediators. Importantly, many cancer cells express PD-L1 as a mechanism of immune evasion. Thus, targeting PD-1 and PD-L1 with antibodies has demonstrated significant therapeutic benefits in clinical trials, especially resulting in activation of the antitumor CTL response, a phenomenon known as immune checkpoint blockade (48).

The structure of PD-L1 in complex with PD-1 has been extensively studied (49). Several X-ray crystal structures are now available, including human PD-L1 alone, mouse PD-1 complexed with human PD-L1, and human PD-1 complexed with human PD-L1 or antibodies. These structures have shown that **pembrolizumab** and **nivolumab** epitopes on PD-1 overlap with part of the PD-L1 binding site. The affinity of these antibodies for PD-1 is in the low picomolar range (50). This is several orders of magnitude stronger than the affinity of PD-L1 for PD-1, estimated in the nanomolar range (51), which suggests that the MOA of **pembrolizumab** and **nivolumab** is through outcompeting PD-L1 for binding to PD-1. Furthermore, **pembrolizumab** and **nivolumab** have been engineered with IgG4 isotypes, which has an important influence in their MOA by reducing toxicity of these antibodies. IgG4 lacks effector functions, such as ADCC and CDC, which may be potentially harmful to the immune cells expressing PD-1 when targeting this ligand with antibodies.

On the PD-L1 side, **atezolizumab**, **durvalumab**, and **avelumab** bind distinct epitopes but all interfere with PD-1 binding (52), preventing the PD-L1/PD-1 interaction. These three checkpoint inhibitors are of the IgG1 class, but the Fcs of **atezolizumab** and **durvalumab** have been modified to eliminate antibody effector functions. **Atezolizumab** is an aglycosylated antibody, whereas **durvalumab** is a Fc-modified triple mutant variant. **Avelumab** is reported to be a non-modified IgG1. Therefore, like with the anti-PD-1 therapeutic antibodies, the MOA of **atezolizumab**, **durvalumab**, and **avelumab** is an interplay between affinity, epitope, and Fc variants.

## Immunogenicity and Human Content

Immunological reactions to biotherapetics involve a complex combination of diverse components not fully yet understood, including product-, disease- and patient-specific factors (53). The lack of standardization in the terminology and approaches used for collecting, analyzing, and presenting immunogenicity data also makes it difficult to find a consensus on immunogenicity results (54).

Nonetheless, pioneering work on the specificity of the immune reactions to peptides (55), prior to determining the amino acid sequence of a protein or its three-dimensional (3D) structure, suggested that the phylogenetic distance between two species is an important factor in eliciting antibodies. Subsequent determination of the amino acid sequence of the first proteins in the early 1960s indicated that the phylogenetic distance between two species is imprinted in the amino acid sequences (56). These differences play a key role in launching specific immune responses against non-self proteins. In fact, diverse *in silico* predictive methods to assess potential immunogenic spots based on the amino acid sequence have been developed and are part of the toolbox used to engineer therapeutic proteins (57). Thus, engineering antibodies with lower immunogenicity has been driven in part by generating amino acid sequences that are as human as possible.

The diversity of antibodies comes from the recombination of diverse germline gene repertoires (25) and somatic mutations generated during the hypermutation process (26). Since the somatic mutations are specific to an immune response and some mutations may be immunogenic in other individuals with a different immunological history and background, it can be assumed that the ideal human antibody from an immunogenicity standpoint should be identical to the genes encoded by the germline repertoire.

The physical maps of the human IGH and IGL genes loci were elucidated in the 1990s, together with an initial estimation of number and germline gene sequences encoding the functional human antibodies (25, 58–60). In the last two decades, more human germline genes from diverse populations have been sequenced and studied. More recently, the application of next-generation sequencing methods to the study the antibody repertoires from diverse individuals have led to the characterization of an increasing number of alleles (61, 62). This information is compiled and curated at the international ImMunoGeneTics information system (IMGT^®^)^1^, with alleles “01” representing the oldest germline genes. The “01” alleles have also been identified in several individuals, thus, perhaps representing the most common human antibody germline genes.

**Figure 3:**
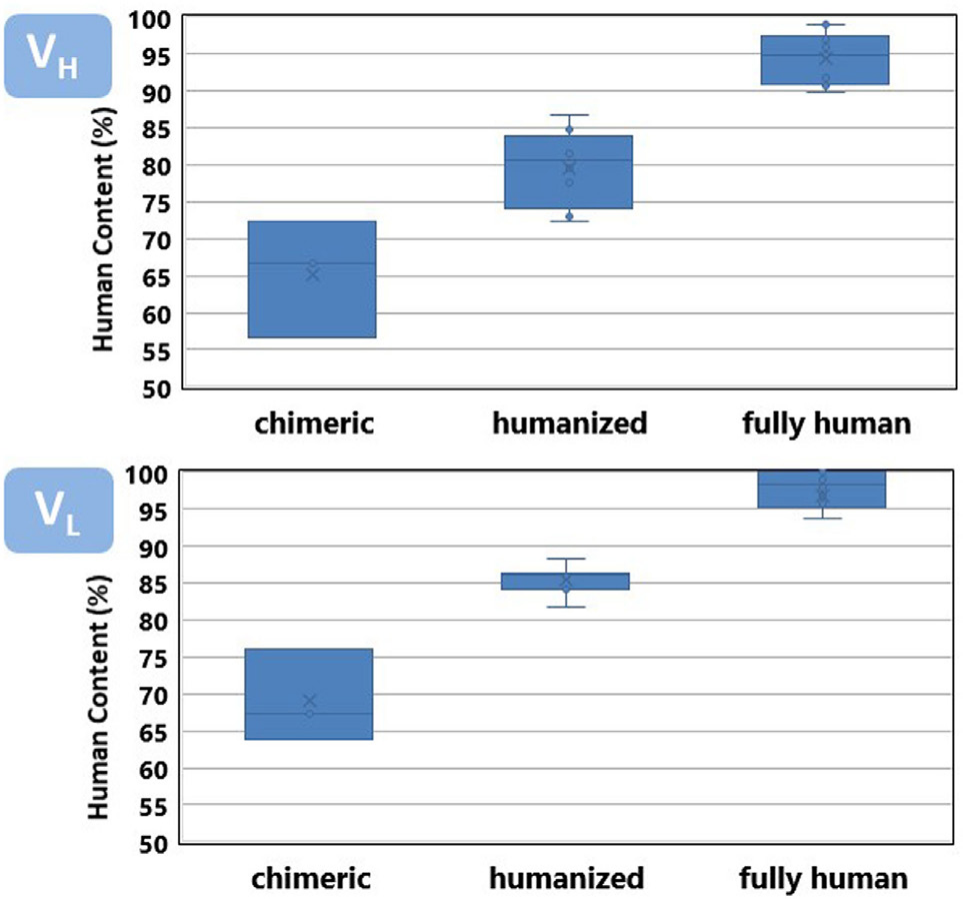
Human content of chimeric, humanized, and fully human antibodies listed in Table 1. See Figure 4 caption for a detailed description of the human content calculation.

Figure 3 shows a comparison of the percentage of identities of the V_H_ and V_L_ regions of the antibodies listed in Table 1 with respect to the closest match in the repertoire of human antibody germline genes compiled at the IMGT. Both V_H_ and V_L_ of the chimeric antibodies have an average around 70% identities with respect to the human germlines, also defined as human content. Chimeric antibodies have a wider variation in the percentage of identities with respect to the human germline genes as well. Humanized antibodies reach an average 85%, whereas, fully human antibodies show 90% or more human content.

Overall, the V_H_ region has a slightly less human content than V_L_. V_H_ leads the interaction of the antibody with its specific target and tends to accumulate more somatic mutations (24), diverging faster from the germline configuration. It poses a higher challenge for antibody engineers to increase the human content of the therapeutic antibodies while preserving the specificity and affinity of the parental, non-human antibody.

The departure from 100% human content observed in fully human antibodies roughly corresponds with the frequency of somatic mutations observed in the antibody human sequences studied by several research groups (63–65). It has been reported that mutations in V_H_ and V_L_ follows an exponential distribution, with as much as 15–20% of the V regions showing no mutations at the amino acid level. Following these sequences in the germline gene configuration, fewer and fewer sequences have an incremental number of mutations. The average number of somatic mutations per human V region observed in diverse samples of sequences product of immune responses has been estimated in around eight and five mutations for V_H_ and V_L_, respectively.

**Figure 4:**
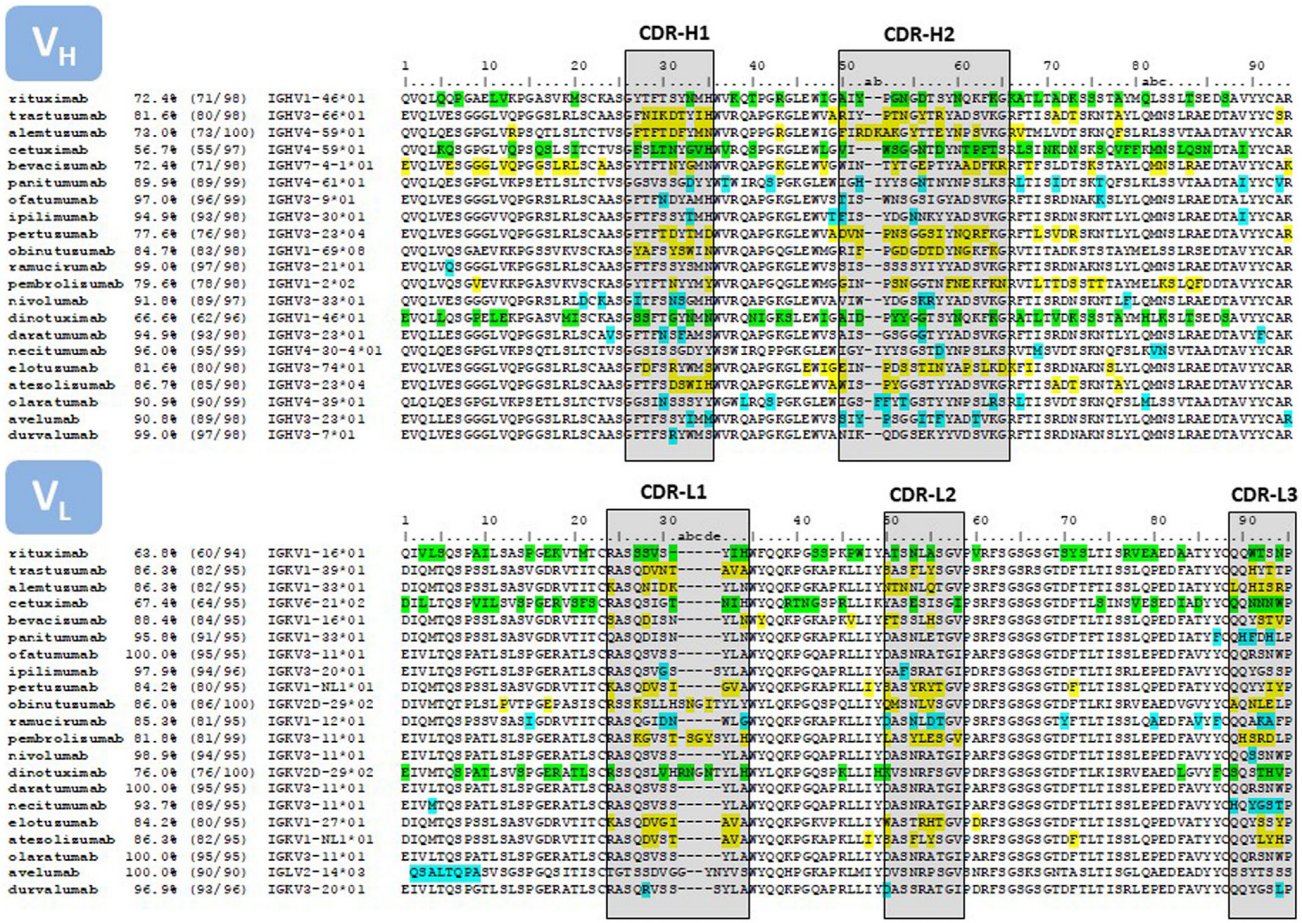
Sequence alignment of the V regions of the antibodies listed in Table 1. Only the amino acids encoded in the IGHV and IGKV genes are reported. The amino acid sequences were taken from DrugBank (66). In those cases where more than one sequence per therapeutic antibody is reported at DrugBank or no sequence was available in this source, we used the sequences compiled by Jain and collaborators (67). The sequences were compared with the repertoire of human germlines compiled at IMGT using IgBLAST (https://www.ncbi.nlm.nih.gov/igblast/) and the percentage of identities as reported in IgBLAST’s output is listed in the second column of the Figure. The third column lists the number of identities divided the length of the amino acid sequence. Some antibodies, in particular the sequence of the chimeric antibodies, matched more than one human germline gene sequence with equal percentage of identities. In these cases, we report the first germline gene in the IgBLAST’s output. In other instances, the antibody sequence matched more than one human germline gene allele with equal number of identities, but with amino acid mismatches at different positions of the V region. In these cases we report the “01” allele. The numbering on top of the sequences corresponds with Chothia’s definition (68). CDRs are delimited with boxes, following Kabat’s definition (69), except at the CDR-H1, which is a combination of Chothia’s and Kabat’s definition. The color code corresponds to mismatches with respect to the closest human germline gene; green, chimeric antibodies; yellow, humanized antibodies; blue, fully human antibodies.

The placement of mutations with respect to the closest germline gene match is shown in Figure 4. Chimeric antibodies show amino acid differences all along the V regions, with a high number of non-human residues in the FR-3 of V_H_ and FR-1 of V_L_. In contrast, the mutations of the humanized antibodies are mostly clustered in the CDRs. Fully human antibodies show very few mutations, with some V regions being in the germline configuration.

### Chimeric Antibodies

After the FDA approval of **rituximab**, two additional chimeric antibodies, **cetuximab** and **dinutuximab**, reached the market for oncology indications. **Cetuximab** was generated by immunizing mice with purified EGFR and replacing the mouse constant domains of the mouse antibody 225 with those of human IgG1. The chimeric molecule, named C225, showed around five-fold higher affinity and increased tumor growth reduction than the parental mouse antibody (70). **Dinutuximab** was developed from a murine antibody specific for GD2 (71). The chimeric molecule (ch14.18), also a human IgG1, showed identical binding as the murine IgG2a antibody but, the ADCC was 50-fold to 100-fold higher than the parental mouse antibody when using human effector cells (72). Therefore, in addition to rendering less immunogenic molecules, chimerization overcame some of the drawbacks of the early murine monoclonal antibodies by generating therapeutic molecules with the same or improved affinity than the parental mouse antibodies but with enhanced effector functions.

### Humanized Antibodies

Although chimeric antibodies were more efficacious and less immunogenic than mouse antibodies, they still elicited a “human anti-chimeric antibody” (HACA) response (73). Thus, to further increase the human content of therapeutic antibodies, in the second half of the 1980s Winter’s group at the Medical Research Council (11) showed that by grafting the CDRs from an antibody into FRs of another antibody, the specificity and affinity of CDR donor antibody can be transferred to the antibody providing the FRs. It was first applied to engineer **alemtuzumab** (74). The parental antibody was a rat IgG2a, called YTH 34.5HL. Its CDRs were grafted into the human V_H_ and V_L_ FRs of the known antibody structures at the time NEW (75) and REI (76), respectively.

In an alternative approach, Queen and collaborators (77) at Protein Design Labs (PDL) humanized daclizumab (Zenapax^®^)—not discussed here as it has no indication in cancer. Daclizumab was humanized by CDR grafting, but the human FRs were selected by maximizing homology between the murine antibody sequence providing the CDRs and the human antibody donating the FRs. In addition, a computer model of the mouse antibody, guided the identification of several murine amino acids in the FRs that interacted with the CDRs or antigen and back mutated those amino acids into the CDR-grafted antibody, thus, improving binding of the final product. Together with the successful humanization of **alemtuzumab** by Winter’s group, these pioneering works laid the foundations for humanization *via* CDR grafting, the humanization method used to engineer all of the humanized antibodies listed in Table 1.

For instance, Carter and collaborators (78) humanized the murine antibody mumAb4D5, which had potential for human therapy due to its anti-proliferative (cytostatic) effect against human breast and ovarian cancer cell lines overexpressing HER2. The CDRs of mumAb4D5 were grafted in consensus human FRs and several mouse residues were incorporated into the FR aiming to retain the affinity of the parental mumAb4D5 antibody. One of the humanized versions of this antibody named humAb4D5-8, later named **trastuzumab**, showed a four-fold increase in HER2 binding affinity compared to the parental mumAb4D5, similar cytostatic activity, and more efficient ADCC against HER2 overexpressing cancer cells. Thus, this study demonstrated that humanization technologies can be helpful not only to increase the human content of Fv region but also to enhance the binding properties of the antibody and its therapeutic efficacy.

**Bevacizumab** (79) and **pertuzumab** (80) were engineered using a similar method. The other humanized antibodies for oncology indications, e.g., **pembrolizumab**, **atezolizumab**, **obinutuzumab**, and **elotuzumab** have also been obtained by grafting non-human CDRs into human FRs, with designed backmutations, which replace human residues by the original mouse residues in the FR to stabilize the CDR conformations and, hence, preserve or improve the affinity of the parental non-human antibody. The number of backmutations varies depending on the source of the FR, namely: mature antibodies (77), consensus FRs (78) and more recently the use of human germline genes (81, 82). The FR selection method, sequence similarity between the parental non-human donor and human acceptor sequences, as well as the affinity for the target are all contributing factors to obtaining humanizing antibodies with higher human content (83).

Although humanized antibodies have more human content than chimeric antibodies they still do not eliminate the possibility of the induction of a “human anti-human antibody” (HAHA) response (84). However, humanization is still broadly used due in part to the accessibility of hybridoma technology to academic laboratories and small biotech companies. Also, the relatively recent expiration of the dominant humanization patents (85, 86) and diversification of humanization methods (83) have contributed to the widespread use of the technology.

While the HAMA response can in principle be directed against the entire antibody and the HACA response against the V regions, the HAHA response is even more focused against the CDRs (see Figure 4). Thus, replacing non-human amino acids in the CDRs with human amino acids has been undertaken by several companies such as Xencor (87), Facet Biotech Corporation (88), and more recently Pfizer (89, 90). For instance, Townsend and collaborators (89) generated libraries of binary substitutions at the CDRs by combining the parental non-human residues with human germline residues at each position and screened the libraries for clones with restored antigen-binding capacity. The resulting antibodies increased the human content by 17–29%, rendering molecules indistinguishable from fully human antibodies. Apgar and collaborators (90) followed a rational approach based on the structure of the antigen-antibody complex and were able to replace 11 out of 26 non-human residues in the CDRs. Thus, it could be expected that due to the accessibility and low cost of the hybridoma technology and commoditization of CDR grafting compounded with CDR humanization rendering antibodies indistinguishable from fully human antibodies, humanized antibodies with a higher human content will reach clinical development in the near future.

### Fully Human Antibodies

Fully human therapeutic antibodies emerged in the 1990s with the development of two technology platforms: human antibody Fv or Fab phage-display libraries (12) and transgenic animals bearing the human antibody repertoire (14). Eleven antibodies listed in Table 1 have been discovered using these platforms. Of note, only three: **necitumumab**, **ramucirumab**, **avelumab** were obtained using phage display. Although phage display was developed prior to transgenic animals, the latter requires less optimization and thus shorter timelines to reach clinical development (91). In addition, since enriching technologies such as phage display are based on *in vitro* selection, the antibody fragments coming out of the selection and screening processes do not undergo the *in vivo* selection process and tend to carry developability liabilities (discussed below).

Two transgenic mice produced the fully human antibodies approved for oncology indications, e.g., Medarex (14) and Abgenix (16). The first therapeutic antibody developed by one of these platforms was **panitumumab**. This therapeutic antibody was obtained using the XenoMouse^®^ (92). **Durvalumab** was discovered using the same platform (93), whereas, the other five antibodies listed in Table 1 were discovered using the Medarex technology. Interestingly, four of the Medarex antibodies share the same V_L_, IGKV3-11*01, with three of them in the germline gene configuration and one (**nivolumab**) with only one mutation at the CDR-L3 (Figure 4). Although the Medarex mouse has only a small fraction of the complete human V_H_ and V_L_ repertoires, it demonstrated that even with limited diversity, the plasticity of V_L_ to pair with diverse V_H_ chains can generate specific and high affinity therapeutic antibodies against unrelated targets. Other transgenic mice have been developed in the last three years including the Kymouse (94) and the Trianni mouse (95). These platforms rely on more diverse repertoires of human antibody genes, which enable the selection of highly diverse human antibodies and circumvent some of the limitations of the early transgenic mice.

Despite the success of transgenic mice as a source of therapeutic antibodies, immunization does not always lead to antibodies with the desired antibody affinity and specificity (96, 97). This is particularly true for conserved epitopes between human and mouse orthologs. Transgenic rats (98) and more recently chickens (OmniChicken) (99) may partially mitigate this limitation. Nonetheless, toxic targets and selection of proteins with specific active conformations in environments of interest for a given MOA are not well suited for an immunization approach and require an alternative solution for antibody discovery.

Phage display opened the possibility of designing and manipulating the repertoire of antibody genes to be used as source of antibodies (24), thus, leading to selection of fully antibodies *in vitro*. Since the discovery process *via* phage display is performed *in vitro* one can also choose the optimal conditions to select for desired biophysical and biochemical properties, target pre-defined epitopes locked in specific conformations, avoid immunodominant epitopes by masking them with other known antibodies and/or focus the selection of rare or cross-reactive epitopes.

For instance, **ramucirumab** was developed starting from three antibodies with identical V_H_ sequence isolated from de Haard and collaborators’ Fab display library (100). These antibodies bound specifically the VEGF receptor 2 (VEGFR2), blocked the VEGF/KDR interaction, and inhibited VEGF-induced proliferation of human endothelial cells and migration of KDR leukemia cells. A new library was built by combining the single V_H_ with a repertoire of naïve V_L_ chains, and diverse and specific V_L_ chains for VEGFR2 were selected. Then, a consensus V_H_:V_L_ pair, termed 1121, was identified after selection tailoring the stringency of the panning conditions to obtain picomolar binders (101).

**Necitumumab** was also isolated from de Haard and collaborators’ library by using A431 carcinoma cells, which express high levels of EGFR. Competition with **cetuximab** for binding to the cell surface generated one clone, termed 11F8, which displayed a dose-dependent inhibitory effect on EGF-stimulated EGFR activation in A431 cells. A comparison of the structures of Fab11F8 with the Fab derived from **cetuximab** (FabC225) both in complex with EGFR, indicated that the epitope of the two Fabs was remarkably similar, but the antibodies having different CDRs, bound EGFR through a set of different interactions (39). **Necitumumab**, the new fully human antibody was thus developed, had similar biological properties to **cetuximab**, but without the disadvantages of a chimeric antibody.

Until relatively recently, phage display technology was controlled by a few companies, holding their technology patents (102). These patents expired in Europe and the United States, thus, allowing the free use of the antibody discovery methodology *via* phage display by academic laboratories and small biotech organizations. As phage display technology has become a commodity, several companies such as BioRad using HuCAL^®^ (103), Distributed Bio *via* SuperHuman synthetic libraries,^2^ and GlobalBio/ADL by means of semi-synthetic ALTHEA Gold Libraries™^3^ are licensing phage display libraries and/or offering discovery services at a relatively low cost without royalty payments.

In addition to phage, other display platforms have been developed, including ribosome (104), bacteria (105), yeast (106), and mammalian (107) display. Each of these platforms has advantages and disadvantages. One of the advantages of yeast display, which is the most commonly used, over phage display is the screening using fluorescence-activated cell sorting. This advantage has proven to be an efficient means to isolate antibodies with very high affinity, e.g., in the low femtomolar range (108). In addition, while phage is limited to the display of antibody fragments such as scFvs or Fabs, yeast enables the display of full IgG antibodies with glycosylation. Since the end therapeutic product is commonly an IgG and its efficacy and toxicity are an interplay between target epitope, affinity, Fc isotype and/or variants and glycosylation, yeast display has become a suitable platform for efficient therapeutic discovery and development (109).

## Developability

As more antibodies have reached the market, and more importantly, many failed to perform in preclinical development and clinical trials, the term “developability” emerged in the 2010s (110, 111). Developability encompasses a set of design principles and experimental methods to assess the potential of antibodies to be further developed or manufactured, formulated, and stabilized to achieve the desired therapeutic effect. For instance, post-translational amino acid modifications such as deamidation of asparagine (N), oxidation of methionine (M), and isomerization of aspartic acid, have been identified as potential developability liabilities (112). Modifications of these amino acids can lead to heterogenicities in the drug and/or lack of potency if these amino acids are involved in the interaction with the target. Other amino acids such as tryptophan (W) can induce aggregation and, thus, immunogenicity or lack of solubility at concentrations required for the therapeutic indication, which impairs the further development of the product. Hence, identification of these amino acids and removal when possible during the early discovery process are now part of the antibody engineering process to increase the success rate of preclinical and clinical development.

On the experimental side, recently Jain and collaborators (67) have assessed the limits of developability of 137 FDA-approved therapeutic antibodies (including those listed in Table 1) as well as those in advanced stages of clinical development, i.e., clinical phase I, II, and III. More favorable biophysical properties were found in approved antibodies. Hence, the biopharma industry has progressively been implementing experimental assessment of biophysical properties in early stages of the discovery campaign to progress molecules that would perform well in preclinical development. This is particularly important for therapeutic antibodies generated *via* phage display, related enriching technologies, and humanized antibodies where the selection process proceed *in vitro* without the filters imposed *in vivo* that tend to select well-behaved molecules when used as therapeutics.

## Modulating Fc-Dependent Effector Functions

As reviewed above, choosing the right IgG isotype is key to achieve the desired MOA. All antibodies compiled in Table 1 are IgG1 except three: **panitumumab**, **nivolumab**, and **pembrolizumab**. The human IgG1 elicits strong effector functions such as ADCC and CDC (Table 2), which have been shown to be an important mechanism to kill cancer cells and has been broadly used in development of anticancer antibodies. However, the importance of the other IgG isotypes such as IgG2 and IgG4, as well as mutations of the Fc region (Table 3) have had a significant impact in the success of cancer-targeting antibodies, and are now an essential part of designing and testing therapeutic antibodies. The current approved Fc-engineered antibodies and further developments in the field of Fc engineering are reviewed in the following sections.

**Table 3:** Examples of human Fc mutations for functional modification.

Function	Effect	Class	Application	Mutations or changes	Reference
ADCC	Enhanced	IgG1	Cancer	S298A/E333A/K334A	(113, 114)
				S239D/I332E	(114, 115)
				S239D/A330L/I332E	(114, 116, 117)
				S298A	(113)
				D280H	(118)
				K290S	(118)
				F243L/R292P/Y300L	(119)
				F243L/R292P/Y300L/V305I/P396L	(119)

	Diminished	IgG1	Cancer	G236A	(120)
			Cancer	K326W/E333S	(121)
			RA	C130S/C136S/C139S/P148S	(122)
			Cancer	C226S/C229S/E233P/L234V/L235A	(123)
			Cancer	S298N, S298V, or S298D	(118)
			Cancer	D265A	(113)
			ID, RA, Cancer	M252Y/S254T/T256E	(124)

		IgG3	Cancer	L234A/L235A/P329S	(125)

		IgG4	Cancer	L235A/G237A/E318A	(126)

ADCP	Enhanced	IgG1	Cancer	G236A	(120)
				S239D/I332E	(114)
				S239D/A330L/I332E	(114)

	Diminished	IgG1	Cancer	C226S/C229S/E233P/L234V/L235A	(123)

CDC	Enhanced	IgG1	Cancer	K326W	(121)
				E333S	(121)
				T256N/A378V/N434Y^a^	(127)
				T256N/A378V/S383N/N434Y^a^	(127)
				P228LI/T256N/A378V/N434Y^a^	(127)
				P230S/N315D/M428L/N434Y^a^	(127)
				K320E/Q386R^b^	(128)

		IgG2	Cancer	K326W/E333S	(121)

	Diminished	IgG1	Cancer	S239D/A330L/I332E	(114)
				C226S/C229S/E233P/L234V/L235A	(123)
				D270A	(129)
				K322A	(129)
				P329A	(129)
				P331A	(129)
				T307A/N315D/A330V/E382V/N389T/N434Y	(127)
				N315D/A330V/N361D/A378V/N434Y	(127)
				E294Del/T307P/N434Y^c^	(127)
				M252Y/S254T/T256E	(127)

		IgG3	Cancer	P329S	(125)

Half-life	Increased	IgG1	ID, RA, Cancer	M252Y/S254T/T256E	(124)
			ID	T250Q/M428L	(130)
			Cancer, AID	N434A	(131, 132)
				L235A/G237A/E318A	(126)
			Cancer	T307A/E380A/N434A	(131)
			Cancer	M428L/N434S	(133)
			Cancer	T307A/N315D/A330V/E382V/N389T/N434Y	(134)
				T256N/A378V/N343Y	(134)
				N315D/A330V/N361D/A378V/N434Y	(134)
				V259I/N315D/434Y	(134)
				P230S/N315D/M428L/N343Y	(134)
				E294Del/T307P/N434Y^c^	(134)

		IgG2	Not disclosed	T250Q/M428L	(130)

		IgG3	ID	R435H	(135)

	Decreased	IgG1	Cancer, AID	I253A	(131)
				P257I/N434H	(136)
			Not disclosed	P257I/Q311I	(136)
				D376V/N434H	(136)

*^a^Produced in YB2/0 cells to yield afucosylated antibodies to enhance ADCC*.

*^b^This construct also includes the T299L mutation that leads to an aglycosylated antibody with reduced ADCC activity*.

*^c^E294Del: residue 294 is deleted in this construct*.

*AID, autoimmune disease; ID, infectious diseases; RA, rheumatoid arthritis*.

*EU numbering is used in all cases*.

### Human IgG Isotypes and Mutations to Alter Effector Functions

ADCC occurs when an antibody simultaneously binds its cognate antigen on the surface of the malignant cell and the Fc region of the antibody binds activating Fc gamma receptors (FcγR) on the surface of an effector cell. This stimulates a signaling cascade within the effector cell that results in the release of cytotoxic granules (see Figure 2) that kill the targeted tumor cell. The activating FcγRs are the high affinity FcγRI (CD64) that is expressed on immune cells such as macrophages, neutrophils, and dendritic cells; the intermediate affinity FcγRIIa (CD32a) that is expressed on macrophages, neutrophils, and Langerhans cells; and the low-affinity FcγRIIIa (CD16a) that is expressed on NK cells, macrophages, and neutrophils (137).

ADCC has been shown to play an important role in the efficacy of many antibodies that target cell surface proteins for cancer therapy (138, 139), especially through the interaction with the FcγRIIIa receptor. The allotype (158V) of FγRIIIa binds IgG with a higher affinity and shows increased ADCC activity compared to the low-affinity allotype (158F) (140). For instance, a correlation exists between the clinical efficacy of **rituximab**, **trastuzumab**, and **cetuximab** administered to cancer patients with a homozygous allotype 158V (141–144). ADCP is a similar effector function that results in phagocytosis instead of the release of granules from the effector cell. Effector cells that are capable of phagocytosis, such as macrophages, can mediate both ADCP and ADCC against targeted tumor cells (145). Increasing the ability of a therapeutic antibody to elicit ADCC and ADCP is advantageous in many applications for cancer therapy where ADCC and ADCP are known to play important roles in eliminating the tumor. An IgG1 named 3F2-3M that is specific for the receptor tyrosine kinase EphA2 and contains three mutations (S239D/A330L/I332E) in the Fc domains demonstrated enhanced ADCC activity against a panel of three EphA2-expressing malignant cells regardless of the FcγRIIIa allotype of the peripheral blood mononuclear cells that were used as effector cells (116). No ADCC was observed against a malignant cell line not expressing EpHA2. These same mutations in a **trastuzumab** variant showed increased ADCC compared to wild-type **trastuzumab** irrespective of HER2 expression levels and FcγRIIIa allotype (114). A different triple mutant S298A/E333A/K334A of **trastuzumab** showed similar effects (113). These studies are important because they suggest that these mutant antibodies can be used effectively in all patients, not just those with certain allotypes. Furthermore, the S239D/A330L/I332E triple mutant of **trastuzumab** and **rituximab** also showed enhanced ADCP (114).

CDC is initiated when the Fc region of an antibody bound to a cancer cell binds the C1q protein, which triggers a cascade of events that culminates in the formation of the MAC that forms transmembrane channels in the cell membrane of the malignant cell leading to cell death. CDC has also been shown to be a mechanism of action of some therapeutic antibodies (138, 139). In these cases, Fc engineering to enhance the CDC activity of an antibody can be advantageous. For example, **rituximab** with single mutations (K326W or E333S) has shown increased CDC (121). It is important to note that an increase in CDC was not always observed with mutations of K326 to other amino acids besides tryptophan (W). This serves as a cautionary note that the amino acid substitution can be critical and should be considered carefully.

For antibodies targeting checkpoint inhibitors such as PD-1/PD-L1, the induction of ADCC, ADCP and/or CDC would potentially lead to the destruction of normal immune cells. For this specific application, the therapeutic antibody functions to block the inhibitory signal leading to the induction of an immune response. For instance, **nivolumab** is a human IgG4 isotype with an S228P mutation, which replaces a serine (S) residue in the hinge region with a proline (P) that prevents Fab arm exchange with endogenous IgG4 antibodies, while retaining the low affinity for activating Fc receptors associated with wild-type IgG4 antibodies. In fact, no *in vitro* ADCC or CDC activity was observed with **nivolumab** in assays using PD-1-expressing activated T cells as target cells (146).

Human IgG1 has also been modified to attenuate the effector functions. For instance, abatacept (Orencia^®^) is an FDA-approved fusion protein consisting of the external domain of human CTLA-4 and the Fc region of human IgG1 that contains four mutations: C130S/C136S/C139S/P148S. Abatacept does not induce ADCC against the human B cell line PM-LCL that expresses CD80 and CD86, which interact with CTLA-4 (122). Note that in case of abatacept binding to the targeted cells occurs *via* CTLA-4 and not *via* a variable region of an antibody. These constructs are known as immunoadhesins because they have an adhesive molecule linked to an antibody Fc fragment. Another example is an Fc-engineered anti-CD70 IgG1 that contains five mutations (C226S/C229S/E233P/L234V/L235A) and shows decreased ADCP activity (123). Furthermore, a human IgG3 targeting the transferrin receptor 1 (TfR1) containing only two mutations (L234A/L235A) showed decreased ADCC activity against TfR1 expressing target cells, an effect that was increased by the addition of a third mutation P329S (125). However, the P329S single mutant showed no effect on ADCC (125).

Similar to ADCC and ADCP, there are instances where the induction of CDC may be harmful. For example, CDC has been associated with injection site reactions (147). Additionally, it has been reported that complement activation may interfere with the induction of ADCC (148). Therefore, for antibodies that have CDC-related toxicities, eliminating the ability of the antibody to elicit CDC would be advantageous. For example, the abatacept fusion protein containing the four mutations C130S/C136S/C139S/P148S that lacks ADCC activity as discussed above, also does not induce CDC against a human B cell line, PM-LCL (122). However, for antibodies in which ADCC is a major antitumor mechanism, a mutant that elicits ADCC but not CDC may be the most efficacious. The C1q binding “epicenter” of human IgG1 has been localized to D270, K322, P329, and P331 (129). Point mutations at any one of these sites in rituximab decreased CDC activity, but not ADCC or binding to FcRn (129). In a human IgG3 targeting TfR1, the P329S mutation abolished CDC activity against TfR1 expressing target cells, but no impaired ADCC was detected with this single mutation (125).

### Glycoengineering

Glycosylation of an antibody can also alter its function. Asparagine-297 (N297) in the C_H_2 domain is conserved among the IgG subclasses (149). Glycosylation at this residue stabilizes the Fc region and keeps it in an open conformation (150). This glycosylation is critical for binding to the activating FcγRs and the induction of ADCC. However, if glycosylation is completely eliminated at this site, the C_H_2 domains collapse inward and binding to FcγRs is lost (149). For example, mutation of this residue to alanine (N297A) eliminates glycosylation and results in an antibody that is unable to bind activating FcγRs (151). Mutation of the nearby residue threonine 299 to leucine (T299L) leads to a similar effect since T299 is considered to be part of the “glycosylation motif” that is important for glycosylation of this residue (N297) (128). Antibodies containing the T299L mutation are unable to elicit ADCC against cancer cells (128).

Modulation of the specific carbohydrate composition at N297 can have the opposite effect and enhance the ADCC activity of the antibody. The affinity of an antibody for the activating FcγRs depends on the composition of the N297 *N*-linked oligosaccharide (152). There are 32 different possible combinations of oligosaccharides that can occur at this site (150). Naturally occurring human IgG and those produced by hybridomas or other common expression systems (including murine myeloma cells such as SP2/0-Ag14, P3X63Ag8.653, and NS0/1; CHO cells; and HEK cells) are usually composed of *N*-acetylglucosamine (GlcNAc) and three mannose residues that form a core carbohydrate. This core is attached to two additional GlcNAc groups to form biantennary branches (150). The addition of galactose at each branch can occur as well as the terminal addition of sialic acid to these galactose molecules. Fucose is often part of the core GlcNAc. This fucose, through steric hindrance, obstructs the interaction of the antibody with the FcγRIIIA (149, 150). Thus, elimination of this fucose molecule while maintaining other forms of glycosylation at this site increases the binding of the antibody to the activating FcγRs, enhancing its ability to elicit ADCC and ADCP (152, 153).

Elimination of the fucose at N297 can be achieved by glycoengineering through various methods to produce afucosylated antibodies that have enhanced ADCC capabilities. Examples are shown in Table 4. The use of an expression system that is unable to attach fucose molecules to the antibody is a common way to produce afucosylated antibodies. *FUT8* encodes the fucosyltransferase enzyme that is responsible for the addition of fucose during protein synthesis. Thus, cells that lack or express low levels of this enzyme produce proteins that lack fucosylation. Rat YB2/0 cells are commonly used for this purpose and many different wild-type and Fc-mutated antibodies that show increased ADCC activity have been produced in these cells (127, 134, 154, 155).

**Table 4:** Glycoengineering examples to enhance ADCC.

Cell line	Species and cell type	Description	Reference
YB2/0	Rat hybridoma (B lymphoblast)	Low natural *FUT8* expression levels	(127, 134, 154)
Ms704	Hamster ovary (CHO/DG44 variant)	*FUT8* knock out	(156)
LEC13	Hamster ovary (CHO variant)	Deficient in GDP-mannose 4,6 dehydratase (GMD)	(157)
CHO	Hamster ovary	Fucosyltransferase-deficient (Biowa Potelligent Technology)	(158)
CHO	Hamster ovary	siRNA knockdown of α1,6 fucosylatransferase	(159)
CHO	Hamster ovary	Bisected, afucosylated carbohydrates by exogenous co-expression of β1,4-*N*-acetylglucosaminyltransferase III and Golgi α-mannosidase II	(160)
CHO	Hamster ovary	Overexpression of GnTIII (GlycoMab Technology)	(161, 162)
HEK293F	Human embryonic kidney	Addition of kifunensine to growth medium to inhibit the *N*-linked glycosylation pathway	(163)
HEK293-EBNA	Human embryonic kidney	Exogenous transient expression of chimeric protein, a fusion between the catalytic domain β1,4-*N*-acetylglucosaminyltransferase III and the localization domains of Golgi-resident enzymes	(164)
Strains B1868/4 and B1868/7	*Tetrahymena thermophile* (ciliate)	Altered glycosylation pattern including lack of fucose	(165)
Lemna Expression System (strain 8627)	*Lemna minor* (plant)	siRNA α1,3-fucosyltransferase and β1,2-xylotransferase	(166)
Strains YAS309	*Pichia pastoris* (yeast)	Expression of *Kluyveromyces lactis* UDP-GlcNAc transporter, α1,2 *Mus musculus* MnsI, β1,2 GlcNAc transferase I, β1,2 *Rattus norvegicus* GlcNAc transferase II, *Drosophila melanogaster* MnsII, *Schizosaccharomyces pombe* Gal epimerase, *Drosophilia melanogaster* UDP-Gal transporter, *Homo sapiens* β1,4 galactosyl transferase	(167)

*CHO, Chinese hamster ovary; HEK, human embryonic kidney*.

Alternatively, siRNA can be used to knock down the expression of this enzyme in commonly used expression systems or exogenous expression of various other glycotransferases can force a specific type of glycosylation at N297, both leading to afucosylation at this residue. For example, overexpression of the glycotransferase β(1,4)-*N*-acetylglucosaminyltransferase III (GnTIII), which catalyzes the addition of bisecting GlcNAc, in CHO cells yielded an IgG with reduced core fucosylation and enhanced ADCC (161). This technology is now known as GlycoMab (162). **Obinutuzumab**, produced using the GlycoMab Technology, was approved by the FDA in 2013 as part of a combination therapy for previously untreated patients with chronic lymphocytic leukemia and later in 2016 as part of a combination therapy for patients with follicular lymphoma that are refractory to or have relapsed after treatment with **rituximab** (168, 169). Metabolic interference with host biosynthesis pathways through the addition of kifunensine, an inhibitor of the *N*-linked glycosylation pathway, to the growth medium during production can also result in antibodies with low fucose levels and enhanced ADCC activity. Other eukaryotic expression systems can also be glycoengineered to produce antibodies with low fucose levels (165–167).

### Mutations to Alter Half-Life

Altering the interaction between FcRn and antibodies may lead to the development of antibodies with higher efficacy due to altered pharmacokinetic and pharmacodynamic properties. Binding of human IgG to FcRn is pH dependent where binding occurs within a pH range of 6–6.5 and release occurs at pH 7.0–7.5 (170–172). IgG is taken up by endothelial cells through pinocytosis, binds the FcRn in endosomes where the pH is 6.0–6.5, and is recycled back to the cell surface where the IgG is released due to the neutral pH of blood. If an antibody does not bind the FcRn, it is routed to the lysosomal pathway where degradation occurs. Thus, binding of IgG to the FcRn is important to prolong the half-life since this interaction rescues the antibody from degradation. For this reason, the FcRn is known as the “*salvage receptor*.”

The serum half-life of human IgG1, IgG2, and IgG4 is 21 days, while that of IgG3 is 5–7.5 days (Table 2). Human IgG3 differs from human IgG1 at residue 435. IgG3 contains arginine (R), while IgG1 contains histidine (H) at this position. Both isotypes show pH-dependent binding to A375 human melanoma cells expressing the human FcRn α chain and that transport of both classes is equally efficient in an *in vitro* transport model using these cells (135). However, when both subclasses are present, IgG1 inhibits IgG3 transport leading to its degradation, which can help to explain its shorter half-life. An R435H variant of IgG3 shows similar half-life to human IgG1 (135).

Increasing the serum half-life of IgG1 can also be advantageous to reduce the frequency of administration of the treatment and enhance efficacy. Residues I253, S254, H435, and Y436 play a relevant role in binding of IgG to FcRn since single alanine (A) substitutions at any of these residues substantially decreases binding to the FcRn (113, 173). Using random mutagenesis and a pH-dependent phage display for selection, numerous mutations have been identified that showed increased binding to human FcRn and increased persistence in human FcRn transgenic mice (127). An N434A mutant of a humanized IgG1 antibody showed increased half-life in cynomolgus monkeys (*Macaca fasicularis*) compared to the wild-type IgG1 antibody targeting the B-cell surface receptor (132). This mutant had increased binding affinity to human and monkey FcRn at pH 6, but negligible binding to FcRn at pH 7.4. Mutant N434W had increased binding at both pH and did not show this increase in half-life, demonstrating the pH-dependent binding of IgG to FcRn (132). The triple mutant M252Y/S254T/T256E (YTE) of an anti-respiratory syncytial virus IgG1 also shows pH-dependent binding including increased binding to human and cynomolgus monkey FcRn at pH 6.0 and efficient release at pH 7.4, which results in a four-fold increase in serum half-life in cynomolgus monkeys (124). Furthermore, this YTE mutant showed increased serum half-life in healthy humans (174).

There may be cases in which a reduction in serum half-life may be advantageous. For example, if the antibody is conjugated to a toxic compound, a longer half-life may lead to more unwanted side effects. For example, the I253A mutant of trastuzumab did not bind FcRn *in vitro* and showed enhanced clearance in human FcRn transgenic mice (131). However, it should be noted that other human IgG1 mutants P257I/Q311I, P257I/N434H, and D376V/N434H specific for tumor necrosis factor-alpha (TNF-α) that showed increased *in vitro* binding to human, cynomolgus, and mouse FcRn at pH 6 and no binding at pH 7.4, paradoxically had increased clearance in CD-1 and C57BL/6 mice (136). The serum half-life of these mutants (P257I/N434H and D376V/N434H) was similar to that of the wild-type IgG1 in cynomolgus monkeys (136).

## Concluding Remarks and Future Developments

In the last 40 years, after the seminal work by Köhler and Milstein, academic laboratories and the biopharmaceutical industry have achieved remarkable progress in the engineering and clinical development of therapeutic antibodies, leading to the approval of 21 antibodies for diverse indications in oncology. V region discovery and engineering platforms have evolved from selecting and developing mouse and rat monoclonal antibodies to engineering chimeric antibodies by joining rodent V regions with human C regions, to humanized antibodies by rodent CDR grafting into human FRs, to fully human antibodies developed *via* phage display or transgenic animals. Fully human antibodies have a higher human content, with some antibodies being in the germline gene configuration and, thus, in principle having minimal immunogenicity.

Although these technology platforms have matured, additional incremental but important improvements are still in progress. For instance, humanization of the CDRs is leading to humanized antibodies indistinguishable from fully human antibodies. Better and more diverse phage display antibody libraries are now available. These libraries are built with variable regions with high expression levels in production cells, highly soluble and more stable than the initial naïve libraries composed of the entire repertoire of human antibodies, some of which may not be developable. Importantly, humanization platforms and phage display methodology are now off patent thus, becoming commodities, which make them accessible to academic laboratories and small biotech companies at a lower cost, fostering more innovation and further exploration of diverse and novel targets. In addition, other platforms such as yeast display have been developed, which allows for efficient selection of high affinity antibodies and full IgG with diverse Fc isotypes and glycosylation. More efficient transgenic mice and transgenic species other than the mouse, such as the chicken, have been generated, which can tackle some of the limitations of the early transgenic mice and expand the possibilities of obtaining antibodies targeting conversed epitopes in human and murine orthologs.

On other hand, in the last 15 years, as more antibodies have been approved for therapeutic settings and many failed to perform in preclinical development and clinical trials, a set of design principles and experimental methods have been implemented to ensure further development or manufacturing of antibodies with therapeutic potential. This has been particularly important for therapeutic antibodies generated *via* phage display and related enriching technologies. Perfecting predictive algorithms to spot in the design phase potential developability issues and applying robust developability experimental methods as early as possible in the antibody discovery phase to select the molecules to further develop should reduce costs and enable more companies to advance fully human antibodies faster and with a higher probability of success in clinical development.

In parallel with the advances in the V region engineering, the Fc has been extensively modified to enhance or attenuate ADCC, ADCP, and/or CDC and thus, tailor the effector functions of therapeutic antibodies to diverse MOAs. Modifications in the residues interacting with the FcRn to extend the half-life of antibodies have been also reported. These modifications are having an impact on dosage and cost of goods, with the ultimate benefit for the treatment of patients. Several of the Fc modifications are being validated in preclinical development and clinical trials and hence it is expected that more therapeutic antibodies with engineered Fc mutations will be approved soon. Beyond IgG, the class of choice for all the approved therapeutic antibodies used for oncologic applications, other classes of antibodies such as IgA (175) and IgE (176) are emerging as new options for cancer therapy. These new options, together with the outstanding progress in the development of antibody engineering methods to modify the V regions should lead to a profound impact in the therapy of cancer in the near future.

## Author Contributions

JA and MP devised the concept of the review and contributed to the overall direction of the manuscript including the general writing, preparation of the figures, and editing processes. JA contributed the sections on antibody engineering. TD-W contributed to the writing and editing of the manuscript with an emphasis on the Fc-engineering section. SP-T and JA contributed the section on the targeted molecules.

## Conflict of Interest Statement

MP is a shareholder of Klyss Biotech, Inc. The Regents of the University of California are in discussions with Klyss Biotech, Inc. to license MP’s technology to this biotech company. The other authors declare that the research was conducted in the absence of any commercial or financial relationships that could be construed as a potential conflict of interest.

## Footnotes

^1^http://www.imgt.org.

^2^http://www.distributedbio.com.

^3^http://www.globalbioinc.com/Services/
